# Australasian sonographers' knowledge, awareness, and attitudes towards the international evidence‐based guidelines for the diagnosis of polycystic ovarian syndrome

**DOI:** 10.1002/ajum.12331

**Published:** 2023-01-18

**Authors:** Alexandra Guscott, Alison Deslandes, Nayana Parange, Jessie Childs

**Affiliations:** ^1^ Allied Health and Human Performance University of South Australia Adelaide South Australia Australia

**Keywords:** attitudes, diagnosis, guidelines, gynaecology, polycystic ovarian syndrome, sonography, ultrasound

## Abstract

**Introduction/Purpose:**

Many guidelines have been utilised to diagnose polycystic ovarian syndrome (PCOS). The most recent are the International Evidence Based Guideline for the Assessment and Management of Polycystic Ovary Syndrome 2018 (2018 IEBG). This study aimed to assess the awareness, knowledge, and attitudes of Australasian sonographers' regarding these guidelines.

**Methods:**

An online cross‐sectional survey was disseminated to sonographers. Qualitative and quantitative questions were asked around awareness, knowledge, and attitudes towards the 2018 IEBG. Statistical and thematic analyses of the results were performed.

**Results:**

Ninety responses were included in the final analysis. Fifty‐two percent (52.2%) of participants were aware of the 2018 IEBG but only 31.1% used it in their workplaces. Fifty‐eight percent (57.9%) of participants correctly identified the sonographic features that suggest PCOS, and 3.5% correctly identified all minimum recommended inclusions for reporting a gynaecological ultrasound for PCOS. Prior to being supplied the 2018 IEBG, 15.8% of participants correctly answered clinical scenario‐based knowledge questions, which increased to 29.4% correctly after being supplied the guideline; however, this difference was not statistically significant. There were no statistically significant associations between demographics and knowledge of the 2018 IEBG.

**Discussion:**

Several areas of confusion surrounding wording and interpretation of the 2018 IEBG were highlighted. Consideration should be given to barriers of implementation and strategies to overcome these.

**Conclusion:**

More education surrounding the sonographic diagnosis of PCOS and the 2018 IEBG is needed. Scanning protocols used amongst sonographers varied, suggesting that inconsistency in sonographic diagnosis may exist. Future reviews of the 2018 IEBG should focus on reducing ambiguity in wording, which may be responsible for some of the varied interpretation of these guidelines.

## Introduction

Polycystic ovarian syndrome (PCOS) is a complex endocrine condition and the mechanism behind it is not completely understood.^1^, ^2^ Common symptoms include hirsutism, acne, menstrual abnormalities, and infertility.^3^, ^4^ Early diagnosis is key in preventing diseases associated with PCOS, such as cardiovascular disease, obesity, diabetes, and some cancers.^5^ The reported prevalence of PCOS is between 4% and 20%, and may differ due to many factors, including the guideline used for diagnosis.^1^, ^2^, ^6^

Polycystic ovarian syndrome remains difficult to diagnose due to varying symptoms, which cause delays in diagnosis and inconsistent treatment.^7^, ^8^ Furthermore, there is wide variation in diagnostic features cited by various published guidelines that are required to be present to confirm a diagnosis of PCOS (Table 1). Additionally, normal limits of diagnostic features have been widely debated and differ with each guideline.^9^

**Table 1:** Diagnostic features of prior (before 2018) guidelines for the diagnosis of polycystic ovarian syndrome (PCOS) produced since 1990.

Name of Guideline	Hyperandrogenism (clinical or biochemical)	Chronic anovulation (clinical)	Polycystic ovarian morphology
1990 National Institute of Health Criteria^9^	Mandatory diagnostic feature	Mandatory diagnostic feature	Not yet defined as a diagnostic feature
2003 Rotterdam Criteria^10^	Potential diagnostic feature	Potential diagnostic feature	Potential diagnostic feature
	Two of the three diagnostic features must be present		
2006 Androgen Excess Society Criteria^9^	Mandatory diagnostic feature	Potential diagnostic feature	Potential diagnostic feature
	Must have hyperandrogenism and at least one other diagnostic feature		
2012 Amsterdam Consensus Statement^9^	Mandatory diagnostic feature	Mandatory diagnostic feature	Mandatory diagnostic feature
2013 Endocrine Society Clinical Practice Guideline^9^	Mandatory diagnostic feature	Mandatory diagnostic feature	Diagnostic feature not included in guideline

In 2003, an international review of diagnostic features was undertaken to produce a consensus on PCOS diagnosis, which led to the publication of the Rotterdam criteria.^10^ In that review, polycystic ovarian morphology (PCOM) as seen on ultrasound was first proposed as a diagnostic feature of PCOS. PCOM was defined as an ovarian volume >10 cm^3^ and/or 12 or more follicles measuring 2–9 mm present on one or both ovaries.^10^ PCOM in the absence of anovulation or hyperandrogenism was not considered diagnostic of PCOS^10^. Despite being based on a single study and not distinguishing between adult and adolescent diagnosis, this definition of PCOM has, until recently, been upheld as the standard of sonographic diagnosis, including in the 2006 and 2012 guidelines.^11^, ^12^, ^13^ Some studies have supported this definition,^4^, ^14^ however, several studies have suggested that advances in technology have resulted in an increased ability to visualise follicles due to increasing resolution of ultrasound machines.^11^, ^15^, ^16^, ^17^ As such, it has been suggested that the threshold of 12 follicles should be increased to as high as 26 follicles per ovary.^11^, ^15^, ^16^, ^17^ Some studies suggested that including sonography in the diagnosis of PCOS may lead to over‐diagnosis of PCOS in people with absent or mild symptoms, particularly when the Rotterdam criteria are used.^5^, ^15^

Two cross‐sectional studies have assessed the prevalence of PCOS using different diagnostic guidelines.^9^, ^15^ These studies found that the prevalence of PCOS differed by 10–21%, depending on which guideline was used for diagnosis.^9^, ^15^ Table 2 lists the guideline used and the percentage of people diagnosed with PCOS according to that guideline. Both studies demonstrated that when the Rotterdam criteria are used, more people are diagnosed with PCOS.

**Table 2:** Percentages of participants diagnosed with PCOS according to different diagnostic guidelines.^9^, ^15^

Guideline used	Percentage of participants diagnosed with PCOS (%)	
	Clark et al. (2014) study	Akgül et al. (2018) study
1990 National Institute of Health criteria	53	78.8
2006 Androgen Excess & PCOS Society Criteria	63	86.5
2003 Rotterdam Criteria	70	100

Key: PCOS, polycystic ovarian syndrome.

In 2018, to combat the conflicting emerging evidence, Monash University, supported by the National Health and Medical Research Council, Centre for Research Excellence in Polycystic Ovary Syndrome, American Society for Reproductive Medicine, and European Society of Human Reproduction and Embryologyconducted an international review of the diagnostic features and guidelines used to diagnose PCOS and produced a new guideline.^18^ This guideline, known as the International Evidence Based Guideline for the Assessment and Management of Polycystic Ovary Syndrome 2018 (2018 IEBG), suggested that features of PCOM be reviewed regularly with advancing technology and they proposed the following sonographic features:

1. Ovarian volume ≥ 10 cm^3^ (height × width × breadth and ensuring the absence of dominant follicles/corpus lutea) and/or
2. Twenty or more follicles on either ovary on high‐quality transvaginal ultrasound (TVUS) and
3. Ultrasound should not be used in people <8 years post‐menarche as PCOM is common at this life stage.

It was also recommended that additional training should be provided for sonographers to ensure accurate reporting of follicle counts.^18^

In Australasia, sonographers are responsible for performing high‐quality ultrasound examinations and as such, play a vital role in diagnosing PCOM, and subsequently PCOS. How much knowledge sonographers' have regarding the diagnostic features of PCOM, according to the 2018 IEBG, and their attitudes towards its use for the diagnosis of PCOM is currently unknown. These factors are important in contributing to the timely and accurate diagnosis of PCOS. Therefore, the aim of this study was to determine sonographer's awareness and knowledge of the 2018 IEBG and their attitudes towards it.

## Methodology

### Study design

Thirty‐five questions were formulated from a literature review on the topic and with the guidance of subject matter experts. Questions included demographic information, awareness, and knowledge of the 2018 IEBG, including the sonographic techniques used to diagnose PCOM. Questions focusing on the attitudes of sonographers towards the role of the 2018 IEBG in the diagnosis of PCOM were also asked. Both open and closed questions were posed, as shown in Table A1 in Appendix A. The data were gathered and analysed anonymously. Incomplete surveys were included in the results. The survey was open for 3 months to allow for sufficient responses.

A sample of convenience was recruited *via* distribution of the survey through social media groups targeted at sonographers and the Australian Society for Ultrasound in Medicine discussion forum. Any accredited or registered student sonographers who perform gynaecological ultrasound in Australia or New Zealand over the age of 18 years were invited to take part. Exclusion criteria included those involved in the development and validity testing of the survey. A sample of 67 responses were required for a 90% confidence interval and 10% margin of error.^19^

### Reliability and validity procedures

The survey was constructed by the principal investigator (AG) in consultation with all authors. A draft survey was provided to a panel of three content experts who ensured the face and content validity of the survey. The content experts assessed the relevance of each question and ensured the wording and terminology were appropriate.^20^ Minor modifications to wording and formatting were implemented, and reliability testing was undertaken. Test–retest reliability was assessed by asking four sonographers to complete the survey twice, 1 week apart, and comparing their answers from both attempts. A percentage agreement was run between the questions with an average agreement of 90% (range 50–100%).

### Statistical analysis

Quantitative results were described with descriptive statistics. The knowledge questions were marked as correct, incorrect, or partially correct by the principal investigator (AG) using the information presented in the 2018 IEBG and checked by a content expert (AD). Fisher's exact test was used to examine any associations between the data and demographics to help inform whether demographic factors influenced the knowledge and attitudes of sonographers. A P‐value of ≤0.05 was considered statistically significant. The program SPSS Statistics for Windows (IBM Corp., Armonk, New York, USA) was used to undertake all statistical analysis.

The open‐ended questions produced qualitative, free text nominal data, which was analysed using a simple thematic analysis undertaken by the principal investigator (AG). This was then checked, and adjustments made by two authors (AD and NP) to ensure trustworthiness of the findings.^21^ Disagreements were resolved *via* verbal discussion until agreement was reached.

### Ethics approval

Ethics approval was obtained from the University of South Australia Human Research Ethics Committee (project number 204169). An online exploratory cross‐sectional survey was conducted using LimeSurvey Version 3.25.12 (Hamburg, Germany) to determine sonographers' awareness, knowledge of and attitudes towards the 2018 IEBG. A participant information sheet was provided, and informed consent was obtained.

## Results

Ninety‐one responses to the survey were received. One respondent was excluded as the person was not from Australia or New Zealand, resulting in 90 complete or partially complete responses being included in the final analysis. Demographic information for participants is outlined in Table 3.

**Table 3:** Demographic information for survey participants. Participants could select the response that was most appropriate for them (N = 90).

Variable	Response	N (%)
Age (years))	18–24	3 (3.3)
	25–34	25 (27.8)
	35–44	25 (27.8)
	45–54	23 (25.6)
	55–64	14 (15.5)
	65+	0 (0)
Gender	Female	80 (88.9)
	Male	10 (11.1)
	Other	0 (0)
State of practice	Queensland	21 (23.4)
	Victoria	18 (20.0)
	New South Wales	29 (32.2)
	South Australia	5 (5.6)
	Australian Capital Territory	4 (4.4)
	Western Australia	4 (4.4)
	Northern Territory	1 (1.1)
	New Zealand	5 (5.6)
	Other/Prefer not to answer	3 (3.3)
Workplace location	Metropolitan area	43 (47.8)
	Regional town	34 (37.8)
	Rural or remote	9 (10.0)
	Combination	4 (4.4)
Clinical environment	Private radiology practice	51 (56.7)
	Private practice within a public hospital	4 (4.4)
	Private practice within a private hospital	6 (6.7)
	Public hospital	20 (22.2)
	Private women's imaging practice	7 (7.8)
	Locum sonographer	1 (1.1)
	Non‐clinical/industry	1 (1.1)
Workplace speciality	General	71 (79.0)
	Obstetric	11 (12.2)
	Breast	2 (2.2)
	Other	4 (4.4)
	Prefer not to say	2 (2.2)
Postgraduate experience (years)	Trainee sonographer	5 (5.60)
	0–2	10 (11.1)
	3–5	11 (12.2)
	6–10	18 (20.0)
	11+	46 (51.1)
Gynaecological ultrasounds per week	0–5	9 (10.0)
	6–10	39 (43.3)
	11–15	117.8 (16/90)
	16–20	14 (15.614/90)
	21+	11 (12.2)
	Prefer not to say	1 (1.1)
Employment status	Full time	42 (46.7)
	Part time	44 (48.9)
	Casual	3 (3.3)
	Unemployed	1 (1.1)

### Awareness of the International Evidence‐Based Guideline for the Assessment and Management of Polycystic Ovary Syndrome 2018

**Figure 1:**
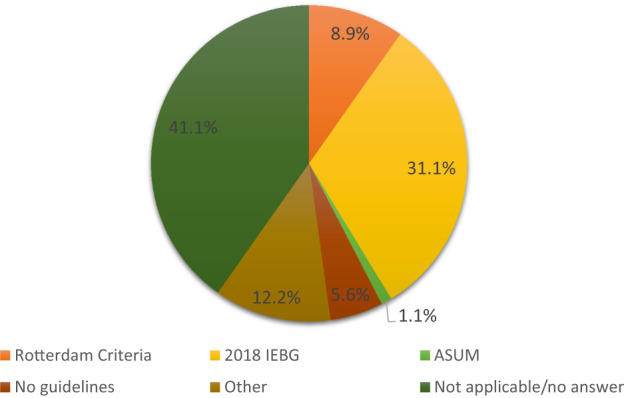
Responses to the question: *Within your workplace, what guidelines are used for the diagnosis of PCOS?* (Participants answered in free text) (N = 90). Key: ASUM, Australasian Society for Ultrasound in Medicine; 2018 IEBG, International Evidence Based Guideline for the Assessment and Management of Polycystic Ovary Syndrome 2018.

Approximately half of the participants (52.2%, n = 47/90) were aware of the 2018 IEBG. Free text responses captured which guidelines sonographers used for the diagnosis of PCOS. Only 31.1% (n = 28/90) used the 2018 IEBG in their workplace, 8.9% (n = 8/90) used the Rotterdam criteria, 5.6% (n = 5/90) had no specific guidelines in their workplace, and 12.2% (n = 11/90) used diagnostic features that were not specific to any published guidelines. Figure 1 shows the guidelines that participants reported using in their workplaces.

### Knowledge of the International Evidence Based Guideline for the Assessment and Management of Polycystic Ovary Syndrome 2018

**Figure 2:**
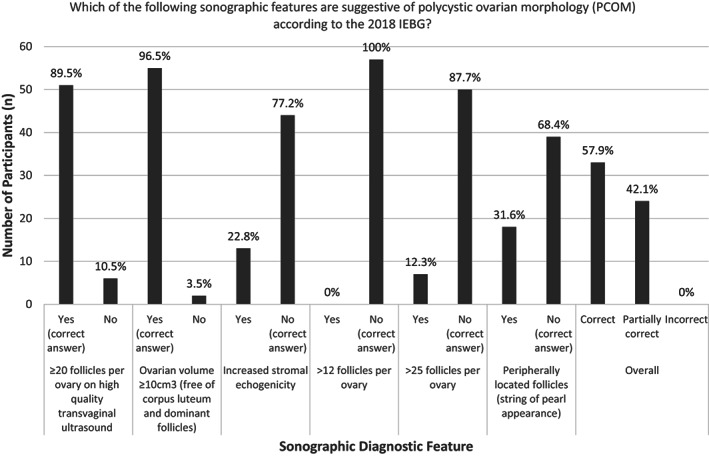
Responses for the question: “Which of the following sonographic features are suggestive of polycystic ovarian morphology (PCOM) according to the 2018 IEBG?”. Respondents to this question (N=57) had the option to select all they believed applied. Key: 2018 IEBG = International Evidence Based Guideline for the Assessment and Management of Polycystic Ovary Syndrome 2018, ≥ = greater than or equal to, 〉 = greater than and * = diagnostic feature does not appear in the International Evidence Based Guideline for the Assessment and Management of Polycystic Ovary Syndrome.

When presented with a list of sonographic features and asked which were suggestive of PCOM according to the 2018 IEBG, 89.5% (n = 51/57) of participants correctly identified ≥20 follicles per ovary on high‐quality TVUS, and 96.5% (n = 55/57) correctly identified ovarian volume ≥ 10 cm^3^ (free of corpus luteum and dominant follicles). However, 22.8% (n = 13/57) of participants incorrectly answered that increased stromal echogenicity was a feature of PCOM and 31.6% (n = 18/57) thought that peripherally located follicles (string of pearl appearance) were also a feature of PCOM according to the 2018 IEBG. Overall, 58.9% (n = 33/57) of participants correctly identified all features, and 42.1% (n = 24/57) were able to identify at least one correct feature. Figure 2 includes all features posed to the participants and their answers.

The minimum recommended inclusions for sonographers reporting an ultrasound for PCOM are to record the last menstrual period, total follicle number per ovary, the three dimensions of each ovary, the endometrial thickness and appearance, the presence of ovarian cysts, corpus luteum, dominant follicles, any other ovarian or uterine pathology along with scan technique (transabdominal, transvaginal) and transducer frequency. Only 3.5% (n = 2/57) of participants selected all correct minimum inclusions. The most commonly missed minimum inclusions for reporting according to the 2018 IEBG was the reporting of any other ovarian and uterine pathology and transducer frequency, with only 14.0% (n = 8/57) and 24.6% (n = 14/57) of participants correctly identifying these minimum inclusions, respectively. The results are presented in Table 4 below.

**Table 4:** Responses to the question: “Which of the following appear in the 2018 IEBG as recommended minimum inclusions for reporting an ultrasound for PCOS?”*.* Respondents to this question (N = 57) had the option to select what they believed applied.

Minimum reporting inclusion	Correct answer	N (%) answering correctly
Last menstrual period	Yes	35 (61.4)
Any medication	No	26 (45.6)
Frequency of transducer (MHz) used	Yes	14 (24.6)
Scan technique (transabdominal, transvaginal, both)	Yes	42 (73.7)
Total follicle number per ovary (2–9 mm)	Yes	39 (68.4)
Three dimensions of each ovary (mm)	Yes	45 (78.9)
Endometrial thickness and appearance (mm)	Yes	22 (38.5)
Presence of ovarian cysts, corpus luteum, dominant follicles (>10 mm)	Yes	40 (70.2)
Ovarian mobility	No	54 (94.7)
Uterine volume (mm^3^)	No	54 (94.7)
Cervical length (mm)	No	57 (100)
Any other ovarian and uterine pathology (mm^3^)	Yes	8 (14.8)
Overall	Correct	2 (3.5)
	Partially correct	55 (96.5)
	Incorrect	0 (0)

Key: 2018 IEBG, International Evidence Based Guideline for the Assessment and Management of Polycystic Ovary Syndrome 2018; PCOS, polycystic ovarian syndrome.

A free‐text question was used to ask at what age it is appropriate to use ultrasound for the diagnosis of PCOM according to the 2018 IEBG. Fifty‐eight percent (57.9%, n = 33/57) of participants answered correctly (over 20 years old or more than 8 years post‐menarche). All participants who answered incorrectly stated ages below 20 years old, without clarifying that this was only if the patient was more than 8 years post‐menarche. Thirty‐nine percent (38.5%, n = 5/13) of those who answered incorrectly stated 18 years old.

The participants were supplied with four clinical scenarios and asked to state whether each was indicative of PCOM according to the 2018 IEBG. The scenarios included information about a patient's age, ovarian volume, and follicle count. These questions were posed twice – once before being supplied the 2018 IEBG and once after being supplied the 2018 IEBG. Before the 2018 IEBG was supplied to the participants, 15.8% (n = 9/57) selected the correct answer for each scenario, while 29.4% (n = 15/51) selected the correct answer for each scenario after being supplied with the 2018 IEBG. Despite the percentage of those answering correctly doubling in number, this was not statistically significant (p = 0.070). Of the 41 people who were aware of the 2018 IEBG, 19.5% (n = 8) answered all the scenario‐based knowledge questions correctly before being given the 2018 IEBG and 31.7% (n = 13) answered correctly after being supplied the 2018 IEBG. Of the participants who were not aware of the 2018 IEBG, none were able to answer all the questions correctly before being supplied the 2018 IEBG, and only one was correct after being supplied the 2018 IEBG (N = 8). These scenarios and participants' answers are detailed in Table 5.

**Table 5:** Knowledge questions assessing which clinical scenario is diagnostic of PCOM on ultrasound. Participants were required to select the best option.

Scenario	Correct answer	N (%) answering correctly
Prior to being supplied the 2018 IEBG (N = 57)		
TVUS, >8 years post‐menarche. LO 9 cc, 23 follicles, RO 9 cc, 19 follicles	Yes	27 (47.4)
TVUS, >8 years post‐menarche, LO 11 cc, 13 follicles, RO 12 cc, 19 follicles	Yes	19 (33.3)
TVUS, 18 yo <8 years post‐menarche, LO 11 cc, 28 follicles, RO 12 cc, 29 follicles	No	35 (61.4)
TAS only, >8 years post‐menarche, LO 9 cc, RO 12 cc and corpus luteum	No	54 (94.7)
Overall	Correct	9 (15.8)
After being supplied the 2018 IEBG (N = 51)		
TVUS, >8 years post‐menarche. LO 9 cc, 23 follicles, RO 9 cc, 19 follicles	Yes	23 (45.1)
TVUS, >8 years post‐menarche, LO 11 cc, 13 follicles, RO 12 cc, 19 follicles	Yes	22 (43.1)
TVUS, 18 yo <8 years post‐menarche, LO 11 cc, 28 follicles, RO 12 cc, 29 follicles	No	44 (86.3)
TAS only, >8 years post‐menarche, LO 9 cc, RO 12 cc and corpus luteum	No	48 (94.1)
Overall	Correct	15 (29.4)

Key: cc, cubic centimetres; 2018 IEBG, International Evidence Based Guideline for the Assessment and Management of Polycystic Ovary Syndrome 2018; LO, left ovary; PCOM, polycystic ovarian morphology on ultrasound; RO, right ovary; TAS, transabdominal ultrasound; TVUS, transvaginal ultrasound, yo, years old.

Evaluating the associations between demographic components and knowledge showed that participants who specialised in obstetrics and gynaecology did not have a significantly better understanding of the 2018 IEBG (p = 0.283). Participants who were aged 55–64 years old were more likely to answer the clinical scenario‐based knowledge questions correctly, however this was not statistically significant (p = 0.088). There was also no statistically significant difference between how many gynaecological ultrasound scans the participants performed per week, and whether the clinical scenario‐based knowledge questions were answered correctly (p = 0.614).

When asked about performing TVUS, 94.7% (n = 54/57) of participants stated they offered this routinely as part of their gynaecological ultrasound examinations, while 5.3% (n = 3/57) only offered this for specific indications such as infertility, PCOS investigation or if requested by the referring doctor. Approximately 87.7% (n = 50/57) of respondents stated that they used a transducer of >8 MHz for TVUS. Ninety‐eight percent (98.2%, n = 56/57) of participants always performed volume measurements of the ovaries during their gynaecological ultrasounds, however, only 47.4% (n = 27/57) of participants worked at a practice that provided a protocol for this. A routine follicle count was performed by 49.4% (n = 27/57) of participants when performing their gynaecological ultrasounds, and 45.6% (n = 26/57) worked at a practice that provided a protocol for performing a follicle count.

### Interpretation and attitudes towards the International Evidence Based Guideline for the Assessment and Management of Polycystic Ovary Syndrome 2018

When asked if participants thought the 2018 IEBG was adequate for the diagnosis of PCOS, there were varying responses. Most participants (58.8%, n = 30/51) either strongly or somewhat agreed that the sonographic features in the 2018 IEBG were adequate for the diagnosis of PCOS, however, 31.4% (n = 16/51) either strongly or somewhat disagreed.

Overall, many participants stated they were satisfied with the 2018 IEBG and thought it provided clarity, insight and was an objective guideline that would help with the diagnosis of PCOS. Eighty‐six percent (86.3%, n = 44/51) of participants said they thought the 2018 IEBG should be used across Australasian ultrasound practices. Many participants believed that implementing the same guidelines was important to provide consistent protocols and reliable diagnoses for patients. One participant supported the idea of standardising practice but was unsure if the 2018 IEBG was the most appropriate guideline. Some participants stated that it was helpful to have more current, evidence‐based guidelines. Others believed that there was ambiguity and gaps in the 2018 IEBG, possibly still leading to misdiagnosis and overdiagnosis, particularly in younger adults and highlighted the need for correlation of sonographic results with biochemical analysis and symptoms.

Several improvements to the 2018 IEBG were suggested, including a statement on the day of the menstrual cycle when ultrasound would be optimal. Another suggestion was to include information regarding the use of hormonal contraceptives (e.g., the oral contraceptive pill or an intrauterine device) and how this may affect the sonographic diagnosis of PCOS. One participant thought a statement regarding the diagnosis of PCOS in asymptomatic people would be helpful. Finally, others suggested features of the ovaries such as peripherally located follicles, or a “bulky” appearance could be suggestive of PCOS and noted if relevant.

## Discussion

The aim of this study was to determine the sonographers' awareness and knowledge of the 2018 IEBG and their attitudes towards the 2018 IEBG for the sonographic diagnosis of PCOS. The key findings indicated that only half (52.2%) of the participants were aware of the 2018 IEBG and only 31.1% of participants reported using them in their practice. Overall, knowledge of the 2018 IEBG was arguably poor. Most participants were aware of the correct follicle count and ovarian volume that were indicative of PCOM. Knowledge of the sonographic features of PCOS according to the 2018 IEBG demonstrated that only 58.9% of participants could identify all the sonographic features indicating PCOM and the remaining 42.1% could identify some. Alarmingly, only 3.5% of participants were able to identify all the minimum reporting inclusions for a gynaecological ultrasound for PCOM while the remaining 96.5% could identify some. This indicates that sonographers had limited awareness of the 2018 IEBG and those who are aware had poor knowledge of the contents. Surprisingly, this was not significantly affected by whether the sonographer worked primarily in obstetrics and gynaecology, although older sonographers had more awareness. Even more surprisingly, this was not significantly affected by whether the participants were supplied the 2018 IEBG, although knowledge did almost double. This was the first study to date assessing sonographer awareness, knowledge, and attitudes towards the 2018 IEBG, providing some insight into possible issues with interpretation of the 2018 IEBG.

Interpretation and knowledge of the guideline was assessed by posing four clinical scenario‐based questions to the participants once before and once after supplying participants with the guideline. Given the lack of awareness and knowledge of the contents of the guideline, it is not surprising that <16% of participants answered these correctly. Almost double the number of sonographers answered the clinical scenario questions correctly after being given the 2018 IEBG to read, compared to before the 2018 IEBG was supplied, raising questions around interpretability. Scenarios one and two were of particular interest, as almost half of the participants answered these incorrectly, even after being supplied the 2018 IEBG. In these scenarios, the patients were more than 8 years post‐menarche. In scenario one, the left ovary had a follicle count of 23, but the follicle counts on the right ovary and both ovarian volumes were within normal limits. In scenario two, both ovaries had an increased ovarian volume, but the follicle counts were normal. According to the 2018 IEBG, both scenarios are indicative of PCOM. This, along with comments made by some participants, indicates that the 2018 IEBG are difficult to interpret. Confusion with these scenarios may be due to the “and/or” statement combining the following terms:

1. Ovarian volume ≥ 10 cm^3^ (height × width × breadth and ensuring the absence of dominant follicles/corpus lutea) and/or
2. Twenty or more follicles on either ovary on high‐quality transvaginal ultrasound.

Another possible area of confusion could be that only one ovary needs to have an increased volume or number of follicles.^18^

When the clinical scenario‐based knowledge questions were posed to the participants, they were deemed correct overall if their answers to all four questions were correct. Participants were deemed as partially correct if some of their answers were correct and some were incorrect. Participants were marked as incorrect if their answers to all the scenario questions were incorrect. Marking of the participants was dependent on the authors' interpretation of the 2018 IEBG being accurate. Another factor that could have contributed to the poor answering of the knowledge questions is that some participants may not have read the 2018 IEBG due to time constraints or other factors.

There is little doubt that having standardised guidelines, such as the 2018 IEBG, is imperative to ensure accurate diagnosis and that terminology used in an ultrasound report, such as ‘PCOM present’ is universal. If the standardised guidelines are unable to be interpreted accurately, however, their usefulness is limited in clinical practice and may result in incorrect diagnosis for patients. In future updates of the 2018 IEBG, consideration should be given to the wording used to ensure there is no ambiguity in the interpretation, especially as they pertain to sonographic diagnosis, to ensure consistency can be achieved when the 2018 IEBG are translated into clinical practice.

The lack of use of the 2018 IEBG in clinical practice is concerning, with many workplaces still using outdated guidelines, relying on information that is based on older equipment, or not using evidence‐based guidelines at all. For example, many sonographers reported using the Rotterdam criteria, which were published in 2003.^10^ Studies suggest this is a widespread issue, with a variety of factors influencing the implementation of new guidelines, including awareness, familiarity, and attitudes towards the guideline, as well as ease of use and credibility of evidence used to inform the guideline.^22^, ^23^, ^24^, ^25^, ^26^ Different guidelines may require individual implementation strategies; however, most sources agree that purely disseminating a guideline is not enough to ensure its implementation.^25^, ^26^ Active learning in the form of education sessions should be provided to sonographers to familiarise them with the guidelines and the evidence base on which they are formed and help translate knowledge into practice.^22^, ^25^ Workplaces should also provide updated protocols, reflecting the new guidelines, with extra time allowed for the ultrasound if required,^22^, ^24^, ^25^ as well as incorporation into university curricula. Feedback from sonographers should be encouraged and acted on throughout the implementation period and beyond.^23^, ^25^, ^26^ Ensuring sonographers are provided with adequate education is also imperative to provide consistency in the diagnosis of PCOM.

Answers to the knowledge questions and some free text comments raised concerns about currency of knowledge of participants. Only 14.0% of participants stated that they would include any other ovarian or uterine pathology, however, it is likely that the participants may have thought this was not relevant to the diagnosis of PCOM rather than sonographers ignoring incidental findings. Approximately a fifth (22.2%) of participants believed that stromal echogenicity was an indicator of PCOM. Almost a third (31.6%) of participants believed that peripherally located follicles (string of pearls appearance) were indicative of PCOM, as well as some participants stating a bulky appearance should be considered for inclusion in the 2018 IEBG. While some studies do support the use of these diagnostic features,^27^, ^28^ they may be subjective and there is not currently enough high‐quality evidence to suggest that these can accurately diagnose PCOM.^18^ Some participants commented that they were unsure if PCOM could be diagnosed in people using hormonal contraception or if there was an optimal time to perform a gynaecological ultrasound during the menstrual cycle. Participants had differing opinions surrounding the age at which a TVUS was appropriate. Many participants stated either 16 or 18 years old. This may be because in some regions, patients can legally consent for medical procedures (e.g. TVUS) at 16 years old and are adults at 18 years old, rather than based on the physiological features that make a TVUS inappropriate for the diagnosis of PCOS at this age. This indicates a need for further and ongoing education of sonographers.

Most participants agreed that the diagnostic features in the 2018 IEBG were adequate for the diagnosis of PCOS. Many were in favour of the concept of consistent protocols for the diagnosis of PCOS to provide more reliability in diagnosis and treatment by using guidelines such as the 2018 IEBG, however, there were suggestions for improvements to the 2018 IEBG. Sonographers will need to be provided with adequate equipment, such as transducers of at least 8 MHz frequency. It is also imperative that sonographers have enough time to include all minimum reporting inclusions, such as ovarian measurement and a follicle count when performing a gynaecological ultrasound for PCOS. Published standardised guidelines for the performance of follicle counting and the measurement of ovarian volume will reduce differences which may exist between centres. Promotion of the 2018 IEBG to provide consistent protocols in the diagnosis of PCOS will likely be beneficial for patients to ensure timely and appropriate treatment. This may need to be promoted by peak sonography bodies and included in educational curriculum to increase awareness of the 2018 IEBG, however, workplaces play the main role in implementation of guidelines, and protocol changes are encouraged.

This study was the first to investigate sonographers' awareness, knowledge, attitudes, and interpretation of the 2018 IEBG at time of publication. A rigorous design was used with an appropriate sample size. Limitations of this study included the small number of sonographers who completed the reliability testing; however, this is not uncommon in a survey design. Other limitations include the possible variation in the way the 2018 IEBG was interpreted and that not all participants answered every question, and sampling bias, as the survey could not be sent to all sonographers in Australian and New Zealand and non‐response bias.^29^ The survey was advertised on several online platforms and reminders were sent out to mitigate these biases, however, there is still a possibility of bias.^29^, ^30^

## Conclusion

Sonographers, generally, were not aware of, and subsequently had poor knowledge of the 2018 IEBG, with few using them in their practice. Some ambiguity or issues interpreting the 2018 IEBG as it pertains to sonographic practice exist which should be considered in future updates of these guidelines. Several improvements of the 2018 IEBG guideline were suggested, such as providing a statement on the effect of contraceptives on a gynaecological ultrasound. Information on the timing of the ultrasound during the menstrual cycle may also aid in planning practice protocols for PCOM diagnosis, along with reviewing the wording of the 2018 IEBG to ensure it can be interpreted accurately. Using consistent protocols is imperative to achieve reliable diagnosis and treatment of PCOS. To do this effectively, interpretation issues of the 2018 IEBG need to be addressed. Other barriers to the implementation of new guidelines also need to be overcome for consistent protocols to be developed and widely used.

## Authorship statement

**Alexandra Guscott:** Conceptualisation (lead); methodology (equal); formal analysis (supporting); writing–original draft (lead), writing–review and editing (equal), **Alison Deslandes:** Conceptualisation (supporting); methodology (equal); writing – review and editing (equal), **Nayana Parange:** Conceptualisation (supporting); methodology (equal); writing – review and editing (equal), **Jessie Childs:** Conceptualisation (supporting); formal analysis (lead); writing – review and editing (equal).

## Funding

No funding information is provided.

## Conflict of interest

Alison Deslandes is the President of the Australasian Society for Ultrasound in Medicine and Nayana Parange is an Associate Editor for the *Australasian Journal of Ultrasound in Medicine*.

## Appendix A

### A.1

**Table A1:** Survey questions posed to participants.

Section of survey	Question	Answer format
Demographics	How old are you?	Multiple choice
	What is your gender?	Multiple choice
	Where do you work?	Multiple choice
	Where is your workplace located?	Multiple choice
	Which best describes your workplace?	Multiple choice
	Which best described your workplace?	Multiple choice
	What specialty is your workplace?	Multiple choice
	How many years of postgraduate experience do you have?	Multiple choice
	How many gynaecological (female pelvic) ultrasounds do you typically perform in a week?	Multiple choice
	What is your current employment status?	Multiple choice
Awareness of the 2018 IEBG	Within your workplace, what guidelines are used for the diagnosis of PCOS? If the guidelines are not used in your workplace, please type N/A	Free text
	Are you aware of the 2018 International Evidence‐Based Guidelines for the Assessment and Management of PCOS?	Multiple choice
	If yes, how did you find out about the content of the guidelines pertaining to ultrasound?	Multiple choice
Knowledge of the 2018 IEBG	Which of the following sonographic features are suggestive of PCOM according to the 2018 IEBG?	Participants could select all options they believed applied
	Which of the following appear in the 2018 IEBG as recommended minimum inclusions for reporting an ultrasound for PCOS?	Participants could select all options they believed applied
	At what age is it appropriate to use ultrasounds for the diagnosis of PCOM?	Free text
Interpretation and knowledge of the 2018 IEBG	An 18 year‐old woman (<8 years post‐menarche) presents for a pelvic ultrasound and a high‐quality TVUSwas performed. The left ovary measures 11 cc and contains 28 follicles. The right ovary measures 12 cc and contains 29 follicles. Is this diagnostic of PCOS?	Multiple choice
	A high‐quality TVUSwas performed on a woman >8 years post‐menarche. The left ovary measures 9 cc and contains 23 follicles. The right ovary measures 9 cc and contains 19 follicles. Is this diagnostic of PCOS?	Multiple choice
	A high‐quality TVUSwas performed on a woman >8 years post‐menarche. The left ovary measures 11 cc and contains 13 follicles. The right ovary measures 12 cc and contains 19 follicles. Is this diagnostic of PCOS?	Multiple choice
	A transabdominal ultrasound only is performed on a woman >8 years post‐menarche. The left ovary measured 9 cc, the right ovary measures 12 cc and contains a corpus luteum. Is this diagnostic of PCOS?	Multiple choice
	When performing a pelvic ultrasound (assuming patient consent), when would you offer transvaginal ultrasound?	Free text
	What frequency transducer is used in your workplace when performing a transvaginal ultrasound?	Free text
	When would you perform volume measurement of the ovaries?	Free text
	Does your practice have a standardised protocol for all sonographers to apply when performing volume measurements of the ovaries?	Multiple choice
	When would you include a follicle count?	Free text
	Does your practice have a standardised protocol for all sonographers to apply when performing follicle counts?	Multiple choice
Interpretation of the 2018 IEBG	2018 IEBG supplied to participants and four scenarios supplied again	
	An 18‐year‐old woman (<8 years post‐menarche) presents for a pelvic ultrasound and a high‐quality TVUSwas performed. The left ovary measures 11 cc and contains 28 follicles. The right ovary measures 12 cc and contains 29 follicles. Is this diagnostic of PCOS?	Multiple choice
	A high‐quality TVUSwas performed on a woman >8 years post‐menarche. The left ovary measures 9 cc and contains 23 follicles. The right ovary measures 9 cc and contains 19 follicles. Is this diagnostic of PCOS?	Multiple choice
	A high‐quality TVUSwas performed on a woman >8 years post‐menarche. The left ovary measures 11 cc and contains 13 follicles. The right ovary measures 12 cc and contains 19 follicles. Is this diagnostic of PCOS?	Multiple choice
	A transabdominal ultrasound only is performed on a woman >8 years post‐menarche. The left ovary measures 9 cc, the right ovary measures 12 cc and contains a corpus luteum. Is this diagnostic of PCOS?	Multiple choice
Attitudes towards the use of the 2018 IEBG in the diagnosis of PCOS	The sonographic features in the 2018 IEBG are adequate for the diagnosis of PCOS.	Likert scale
	What is your opinion of the 2018 IEBG?	Free text
	Do you think the 2018 IEBG should be routinely used across all sonography practices in Australia and New Zealand?	Multiple choice with space for free text comment
	Is there anything you think should be included in sonographic guidelines to diagnose PCOS?	Free text
	Do you have any other comments about the use of ultrasound in the diagnosis of PCOS?	Free text

Key: cc, cubic centimetres; 2018 IEBG, International Evidence Based Guideline for the Assessment and Management of Polycystic Ovary Syndrome 2018; N/A, not applicable; PCOM, polycystic ovarian morphology; PCOS, polycystic ovarian syndrome; TVUS, transvaginal ultrasound; >, greater than; <, less than.
